# Improving the Calibration of Low-Cost Sensors Using Data Assimilation

**DOI:** 10.3390/s24237846

**Published:** 2024-12-08

**Authors:** Diego Alberto Aranda Britez, Alejandro Tapia Córdoba, Princy Johnson, Erid Eulogio Pacheco Viana, Pablo Millán Gata

**Affiliations:** 1 Department of Engineering, Universidad Loyola Andalucía, Avda. de las Universidades, s/n, Dos Hermanas, 41704 Seville, Spain; atapia@uloyola.es (A.T.C.); epacheco@uloyola.es (E.E.P.V.); pmillan@uloyola.es (P.M.G.); 2 School of Engineering, Liverpool John Moores University, Liverpool L3 3AF, UK; p.johnson@ljmu.ac.uk

**Keywords:** soil moisture sensors, calibration, data assimilation

## Abstract

In the context of smart agriculture, accurate soil moisture monitoring is crucial to optimise irrigation, improve water usage efficiency and increase crop yields. Although low-cost capacitive sensors are used to make monitoring affordable, these sensors face accuracy challenges that often result in inefficient irrigation practices. This paper presents a method for calibrating capacitive soil moisture sensors through data assimilation. The method was validated using data collected from a farm in Dos Hermanas, Seville, Spain, which utilises a drip irrigation system. The proposed solution integrates the Hydrus 1D model with particle filter (PF) and the Iterative Ensemble Smoother (IES) to continuously update and refine the model and sensor calibration parameters. The methodology includes the implementation of physical constraints, ensuring that the updated parameters remain within physically plausible ranges. Soil moisture was measured using low-cost SoilWatch 10 capacitive sensors and ThetaProbe ML3 high-precision sensors as a reference. Furthermore, a comparison was carried out between the PF and IES methods. The results demonstrate that the data assimilation approach markedly enhances the precision of sensor readings, aligning them closely with reference measurements and model simulations. The PF method demonstrated superior performance, achieving an 84.8% improvement in accuracy compared to the raw sensor readings. This substantial improvement was measured against high-precision reference sensors, confirming the effectiveness of the PF method in calibrating low-cost capacitive sensors. In contrast, the IES method showed a 68% improvement in accuracy, which, while still considerable, was outperformed by the PF. By effectively mitigating observation noise and sensor biases, this approach proves robust and practical for large-scale implementations in precision agriculture.

## 1. Introduction

Accurate soil moisture monitoring is crucial for effective irrigation management and sustainable farming practices. Reliable moisture data allow farmers to optimise water use, reduce wastage and ensure that crops receive the right amount of water at the right time. This is essential not only for maximising crop yields but also for conserving water resources, particularly in water-scarce regions.

Soil moisture sensors are widely used to monitor soil water content, providing data for decision making in precision agriculture. These sensors enable real-time monitoring, allowing farmers to make informed decisions on irrigation scheduling. Accurate soil moisture data help avoid both over- and under-irrigation, which can cause crop stress, reduced yields and inefficient use of water and energy [1].

Low-cost soil moisture sensors, particularly capacitive ones, play an important role in making monitoring systems economically viable for farmers. These sensors measure soil moisture by detecting changes in the soil’s dielectric constant, which varies with water content. Due to their affordability and ease of deployment, capacitive sensors hold promise in precision agriculture to help optimise irrigation and improve crop yields [2].

Although some sensor manufacturers provide calibration equations relating sensor output to soil moisture content, these equations are not universal and are heavily influenced by soil type and environmental conditions [3]. Calibration involves deriving the appropriate mathematical equations, or calibration curves, that correlate raw sensor measurements with actual soil moisture levels [4]. Regardless of sensor type, calibration is essential for accurate performance, especially in dynamic environmental conditions where variations in soil properties and climate introduce significant uncertainties.

A widely employed calibration method consists in establishing an experimental correlation between raw sensor measurements in soil samples and their known moisture content, being this last typically determined through direct measurement methods, such as oven-drying [2,4,5]. However, this is a time-consuming process, particularly challenging due to soil spatial variability. Different soil types have unique physical and chemical properties, such as texture, organic matter content, electrical conductivity and mineral composition, making the relationship between sensor output and actual soil moisture highly soil-specific [6,7]. As a result, researchers often need to establish specific calibrations for each soil type or profile within their study area. Additionally, factors like soil compactness, inadequate contact between soil and sensor, vegetation and localised heterogeneity can cause the sensor to produce different outputs for the same soil moisture level [8]. Consequently, a suitable calibration method must be specific to both soil and site. Conventional calibration methods are inadequate for large sensor networks because they are time-consuming, labour-intensive and fail to account for changing environmental conditions. Therefore, the focus is on fast and agile methods that maintain accurate calibration while offering flexibility and efficiency to low-cost sensors.

Data assimilation offers a robust approach to calibrating soil moisture sensors by integrating real-time observational data with model simulations to improve prediction accuracy. By continuously updating model parameters, this method effectively captures the spatio-temporal dynamics of volumetric water content (VWC) in the soil. This approach mitigates uncertainties by combining the real-time insights of observational data with the broader contextual understanding provided by model simulations. Despite challenges such as sparse or noisy data and the dependency of models on accurate inputs, data assimilation demonstrates adaptability to varying soil properties and environmental conditions [9,10].

Another technique for enhancing sensor measurements is data fusion, which integrates information from multiple sensors or sources to produce more accurate, consistent and comprehensive datasets [11]. Data fusion primarily aims to merge data from various sources to improve spatial resolution and measurement accuracy, proving particularly advantageous when multiple sensors are deployed. While multiple sensors can increase data volume and potentially strengthen the robustness of data assimilation, they are not strictly necessary. Data assimilation can function effectively with data from a single sensor, leveraging dynamic model simulations to provide valuable real-time updates and improve predictive performance [12].

A large number of studies based on the Kalman Filter (KF) can be found in the literature, together with its derivatives, such as the Extended Kalman Filter (EKF) and Ensemble Kalman Filter (EnKF) for soil moisture data assimilation. These methods, which focus on updating model states with new observations, have been particularly relevant in the integration of satellite and in situ sensor data into hydrological and land surface models [13,14,15]. However, a major limitation of the EnKF lies on its inability to consistently ensure that the updated parameters and state variables adhere to the non-linear constraints of the Richards equation, which governs water flow in unsaturated soils [16]. Another critical limitation of the Kalman Filter-based methods is its sequential nature, which processes observations one time step at a time and does not fully utilise the historical data available. By not accounting for the entire history of data, they may miss important temporal correlations that could improve the understanding and prediction of soil moisture levels.

The Particle Filter (PF), on the other hand, is a widely used method for soil moisture data assimilation, valued for its ability to handle highly non-linear processes and non-Gaussian distributions commonly encountered in soil moisture dynamics. The PF works by forecasting the evolution of particles over time and resampling them based on their alignment with observed data. Each particle represents a possible state of the system, and the weights assigned to each particle reflect the likelihood of that state given the observations. There are studies in the literature that employ particle filter-based methods, which offer promising results in the assimilation of soil moisture sensor data. These methods are used either with sensor data or satellite data for the estimation of hydraulic parameters, water consumption or to predict moisture levels [17,18,19].

The use of the Iterative Ensemble Smoother (IES) in data assimilation for soil moisture sensors has gained significant traction in recent years due to its ability to improve the accuracy of soil moisture estimations. The IES is an advanced data assimilation technique designed to refine model predictions by iteratively updating model parameters using observed data. It belongs to the family of ensemble-based methods, where an ensemble of model realisations is generated to represent the uncertainty in model states and parameters. Unlike the Ensemble Kalman Filter (EnKF), which sequentially updates model states in time, IES focuses on adjusting the entire ensemble simultaneously by assimilating all available observations at once.

A key factor for the successful implementation of data assimilation in sensor calibration is the ability to account for potential biases in both the model parameters and the observations [20], as these deviations can affect the correlation between the measurements and the model parameters. In [21], which serves as an inspiration for this study, the authors perform data assimilation while considering a linear bias in the observations, simultaneously correcting the model parameters. The study focuses on data from Frequency Domain Reflectometry (FDR) sensors and meteorological data, employing methods such as the Ensemble Kalman Filter (EnKF) and Iterative Ensemble Smoother (IES) for data assimilation. While this approach shows promising results for the calibration of FDR sensors, there are limitations when applying it in practical settings. For example, in the case of a farm, the primary source of water is typically supplied by an irrigation system rather than rainfall, and the amount of water provided at each irrigation point is often unknown. Furthermore, the study does not provide validation results using reference sensors to verify the calibration. Finally, one important consideration when updating the model parameters is ensuring that they adhere to certain physical constraints, particularly those related to the hydraulic parameters of the specific type of soil, which is not addressed in this work.

In data assimilation, the main objective is to reduce the difference between observed data and the model’s output by adjusting the model parameters. These parameters are typically bounded by uncertainties and are influenced by observational data. However, the feasible range for these parameters may be further constrained by physical laws, such as conservation principles or thermodynamic limits. Ignoring these constraints can lead to physically unrealistic parameter values, which, although they may improve model accuracy in specific cases, can cause broader failures or non-physical results in different conditions [22]. To address the last mentioned limitation, recent research has focused on integrating constraints in data assimilation techniques. Imposing constraints ensures that the updated states and parameters reflect physical realities and adhere to known properties of the soil or other relevant environmental factors. By leveraging fundamental soil properties and constraints, it is possible to effectively guide the assimilation process towards more robust and applicable outcomes. In [22], the authors introduce a Constraint Iterative Ensemble Smoother (CIES) specifically designed to enforce both linear and non-linear constraints on model parameters. This method partitions the parameter space into subdomains, enabling a multi-solution search for history matching. The algorithm ensures that the optimisation process adheres to a set of constraints by projecting solutions onto a feasible domain at each iteration.

The increasing demand for efficient water management, particularly in agricultural irrigation, underscores the importance of accurate soil moisture monitoring. Precise soil moisture data are critical for informed real-time decision-making in irrigation practices. In this context, the proper calibration of low-cost sensors, such as capacitive soil moisture sensors, is crucial. However, conventional calibration techniques often present significant limitations in field applications, as they typically require expensive and sophisticated infrastructure, which restricts their practicality in small-scale agricultural environments like drip irrigation systems. This paper addresses these limitations by proposing an innovative data assimilation-based method for calibrating low-cost soil moisture sensors. Unlike previous studies that have largely focused on theoretical approaches in controlled environments or remote monitoring settings, our study is uniquely oriented towards practical applications in automated drip irrigation farms using low-cost capacitive sensors. This practical focus not only enhances the effectiveness of the calibration process compared to conventional techniques but also offers a more accessible and precise methodology suitable for widespread agricultural use.

A key innovative aspect of our method is the incorporation of constraints based on fundamental soil knowledge, integrating physical limits and value ranges specific to the soil’s characteristics into the calibration process. These constraints enhance the reliability of the calibration by accounting for the unique properties of the agricultural environment where the sensors are deployed.

Furthermore, and most notably, the study presented in this paper contributes to the field by validating the calibration using a high-precision reference sensor, specifically the ThetaProbe. To the best of our knowledge, this direct validation with a reference sensor in the context of calibrating low-cost soil moisture sensors has not been systematically addressed in previous research. This field validation, conducted on a drip irrigation farm in Dos Hermanas (Spain), provides robust empirical evidence demonstrating the improved accuracy of low-cost sensors achieved through our proposed methodology.

The rest of this paper is structured as follows: Section 2 describes the proposed approach employed in the study, including the assimilation processes. Section 3 presents the results and discusses the performance of the method at both synthetic and field cases. Section 4 discusses the impact of calibration on the performance of an automated irrigation system. Section 5 concludes the paper, and Section 6 outlines potential future research directions.

## 2. Proposed Approach

This section outlines the proposed approach for calibrating soil moisture sensors using data assimilation techniques. The methodology integrates observed VWC time series data from sensors with a numerical soil water flow model to iteratively update and refine model parameters. The process begins with the numerical simulation of soil water flow, accounts for measurement biases in sensor data, constructs a comprehensive parameter matrix and employs data assimilation methods to refine these parameters. The application of parameter constraints ensures that the updated parameters remain within physically plausible ranges.

### 2.1. Overview of the Data Assimilation Process

**Figure 1:**
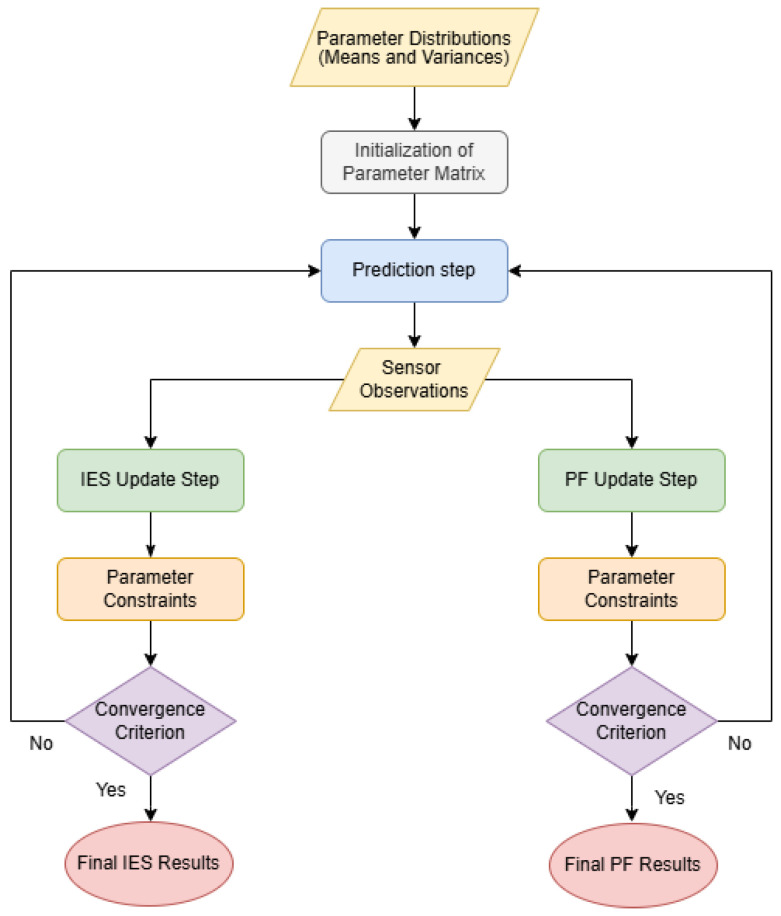
General scheme of the data assimilation process for sensor calibration.

The overall data assimilation process, illustrated in Figure 1, integrates observed sensor data with model simulations to iteratively calibrate soil moisture sensors. The workflow consists of several key steps: initialisation, prediction, update, application of parameter constraints and convergence assessment. These steps enable the implementation of the IES and PF data assimilation methods used in this study. Both methods employ multiple realisations of the system’s state and parameters. In the context of the IES, these realisations are referred to as ensemble members whereas, in the PF, they are commonly known as particles. For clarity and to align with standard terminology, we will use ensemble member when discussing the IES and particle when discussing the PF. There are two primary inputs in the core of this process:

- Parameter Distributions: These form the statistical basis for initialising the parameter matrix $X_{0}$, ensuring that ensemble members accurately represent the system’s variability. The parameters include soil hydraulic properties (e.g., those of the van Genuchten model used in this study), the irrigation flow parameter and calibration coefficients.
- Observed Data (Sensor Time Series): The VWC measurements collected over time from soil moisture sensors provide real-time information about soil moisture dynamics. These data are incorporated during the update step to correct and refine model predictions, ensuring that the assimilation process reflects actual field conditions.

Using the parameter distributions, the parameter matrix is initialised to encapsulate model parameters, calibration coefficients and irrigation flow magnitudes. The prediction step propagates the parameter ensemble through the numerical soil water flow model, simulating VWC over time for each ensemble member. These simulations generate predicted VWC time series, which are subsequently compared with the observed sensor data.

In the update step, the data assimilation methods adjust the parameters in the parameter matrix based on the discrepancies between the model predictions and the observed data. In the IES, ensemble members are statistically adjusted to refine parameter estimates, whereas in the PF, they are resampled and reweighted, based on their likelihood of matching the observed data. Parameter constraints are then applied to ensure physical plausibility, and convergence is assessed to determine whether further iterations are necessary.

The ultimate objective of the data assimilation methods is to obtain the calibration parameters required to correct sensor measurements, thereby enhancing the accuracy of soil moisture estimates. These calibration parameters represent the final results of the assimilation process and are crucial for adjusting the biased sensor readings to better reflect the true soil moisture content.

### 2.2. Water Flow Model

The amount of water in the soil is usually quantified in terms of the VWC, θ, and is formally defined as follows:

(1) $$\theta =\frac{V_{w}}{V_{wet}}$$

where $V_{w}$ and $V_{wet}$ represent, respectively, the volume of water and the total volume of the soil sample. It serves as a critical factor in the equations that depict water flow in porous media, including Richard’s equation. This equation is a mathematical representation of the movement of water through saturated and unsaturated soils, and is expressed as follows:

(2) $$\frac{\partial \theta }{\partial t}=\frac{\partial }{\partial z}\left[K\left(h\right)\left(\frac{\partial h}{\partial z}+1\right)\right]$$

where *z* is the depth, *t* is time, *h* is the water pressure head and *K* is the unsaturated hydraulic conductivity which describes the ease with which a fluid can move through the porous space. The van Genuchten model [23] describes the relation between pressure head and the VWC and is given by the equation

(3) $$\theta \left(h\right)=\theta_{r}+\frac{\theta_{s}-\theta_{r}}{{\left[1+|ah{|}^{n}\right]}^{m}}$$

where $\theta_{r}$ is the residual VWC, $\theta_{s}$ the saturated VWC, α is the parameter related to the inverse of the air entry suction, *n* is the parameter related to the pore-size distribution and *m* is 1−1/n. It also describes the relation between the hydraulic conductivity and the relative saturation $S_{e}$:

(4) $$\begin{array}{ccc} K(h) & = & K_{s}S_{e}^{0.5}\left[1-{\left(1-S_{e}^{\frac{0.5}{m}}\right)}^{m}\right] \end{array}$$

(5) $$\begin{array}{ccc} S_{e} & = & \frac{\theta -\theta_{r}}{\theta_{s}-\theta_{r}} \end{array}$$

where $K_{s}$ is the saturated hydraulic conductivity. The Hydrus 1D software, version 4.17, [24] is used to solve these equations, providing a robust tool for simulating water flow and solute transport in variably saturated media. By inputting the soil hydraulic parameters, Hydrus 1D can numerically solve the Richards equation, which governs unsaturated water flow, and account for various boundary and initial conditions. This capability allows researchers to predict moisture movement and distribution within the soil profile accurately.

### 2.3. Measurement Bias

Due to multiple factors such as the compactness of the soil, vegetation, the presence of heterogeneity, poor contact between the soil and the measuring surface of the sensor or accumulation of moisture on it, the VWC measurements could experience a bias represented by equation

(6) $$\theta_{obs}=a\theta_{true}+b$$

where $\theta_{obs}$ is the sensor observations, $\theta_{true}$ is the actual VWC, *a* and *b* are the bias coefficients. These coefficients are unique to each sensor but assumed constant over time, unless another external factor intervenes.

The data assimilation methods aim to estimate these calibration coefficients *a* and *b* accurately. Once these coefficients are estimated from the data assimilation process, they can be used to correct the sensor measurements, resulting in an improved time series of estimated true VWC. By applying the inverse of the measurement bias equation, the corrected VWC estimates $\theta_{c}$ are calculated as follows:

(7) $$\theta_{c}=\frac{\theta_{obs}-b^{a}}{a^{a}}$$

where $a^{a}$ and $b^{a}$ are the estimation of calibration parameters. This correction adjusts the sensor observations $\theta_{c}$ to account for the identified biases, yielding more accurate estimates of VWC.

### 2.4. Parameter Matrix

The parameter matrix $X_{k}$ is central to the data assimilation process. This matrix encapsulates all parameters to be estimated or updated at each iteration *k* and it is defined as follows:

(8) $$X_{k}={\left[\Theta_{r,k}^{T}\;\Theta_{s,k}^{T}\;A_{\alpha ,k}^{T}\;K_{s,k}^{T}\;N_{k}^{T}\;K_{u,k}^{T}\;A_{k}^{T}\;B_{k}^{T}\right]}^{T}$$

where $X_{k}$ is a matrix of dimensions m×r, where *m* denotes the number of parameters, and *r* represents the number of ensemble members. In this study, m=8, as the parameter matrix includes the following:

- $\Theta_{r}$ and $\Theta_{s}$ are vectors of dimensions 1×r representing the residual and saturated water content parameters ($\theta_{r}$ and $\theta_{s}$), respectively, for each ensemble member.
- $A_{\alpha }$ and *N* are vectors of dimensions 1×r representing the soil pore size distribution parameters (α and *n*), respectively, for each ensemble member.
- $K_{s}$ is a vector of dimension 1×r representing the saturated hydraulic conductivity ($k_{s}$) for each ensemble member.
- $K_{u}$ is a vector of dimension 1×r representing the irrigation flow ($k_{u}$) for each ensemble member.
- *A* and *B* are vectors of dimensions 1×r representing the calibration coefficients (*a* and *b*), respectively, for each ensemble member.

The ensemble size *r* corresponds to the number of Monte Carlo realisations, with each realisation representing a possible state of the system or parameter set. This enables the model to capture uncertainty in parameter estimates effectively. In the context of the IES method, *r* defines the number of ensemble members used to approximate covariances and update parameters iteratively. For the PF method, *r* specifies the number of particles used to approximate posterior distributions and perform resampling. During the prediction stage, which is common to both methods, the model is propagated *r* times, corresponding to each realisation (ensemble member or particle), i.e., each column of the parameter matrix $X_{k}$, resulting in a set of simulated VWC time series. As a result, the ensemble size *r* has a direct influence on the computational cost of the data assimilation process, with larger *r* values requiring a greater number of model simulations.

The parameter $k_{u}$ is used to regulate the irrigation flow as follows:

(9) $$U_{a}=k_{u}\cdot U$$

where *U* is a control vector of size 1×q, containing ones at the timestamps when irrigation is activated and zeros when it is not, with *q* is the total number of VWC observations over time as simulated by the model. This allows the model to dynamically adjust irrigation flows based on the simulation needs.

During the initialisation phase, common to both the IES and PF methods, the parameter matrix $X_{k}$ is constructed. Each column of $X_{k}$, denoted as $x_{j,k}$, represents the parameter vector for ensemble member *j* at iteration *k* in the IES method, or particle *j* in the PF method. These columns are sampled from assumed parameter distributions, ensuring that the ensemble (or set of particles) reflects the uncertainty in the system’s initial conditions.

The parameters $\theta_{r}$, $\theta_{s}$, α, *n* and $k_{s}$ are assumed to follow log-normal distributions. Their corresponding log-normal parameters ($\mu_{\log}$ and $\sigma_{\log}$) are derived from the mean (μ) and variance ($\sigma^{2}$) of their normal distributions using the following transformations:

$$\mu_{\log}=\ln\left(\frac{\mu^{2}}{\sqrt{\sigma^{2}+\mu^{2}}}\right),\;\sigma_{\log}^{2}=\ln\left(1+\frac{\sigma^{2}}{\mu^{2}}\right)$$

The parameters $k_{u}$, *a* and *b*, which are assumed to follow normal distributions, are directly sampled using their specified means and variances.

Each parameter is initialised by drawing random values from its respective distribution:

- For parameters $\theta_{r}$, $\theta_{s}$, α, *n*, and $k_{s}$ (log-normal distributions):
  $$\mathrm{Parameter}\;\mathrm{value}=\exp(N\left(\mu_{\log},\sigma_{\log}\right))$$
- For parameters $k_{u}$, *a*, and *b* (normal distributions):
  Parametervalue=N(μ,σ)

### 2.5. Iterative Ensemble Smoother

The IES is a data assimilation technique used to improve the estimation of state variables and parameters in dynamical systems. Unlike traditional smoothing methods, which typically require the storage of all past observations and states, the IES provides a more efficient and computationally feasible approach by iterating on the ensemble of state estimates. This method leverages an ensemble of model states to approximate the probability distributions of the states and parameters, updating them iteratively based on historical observations.

The IES operates iteratively, with each iteration *k* consisting of a prediction step followed by an update step. During the prediction step, the soil water flow model (Hydrus 1D) is simulated for each ensemble member using parameters from $X_{k-1}^{a}$, the parameter matrix adjusted after assimilation at the previous iteration. This yields forecasted VWC time series $D_{k}^{f}$ with dimension of q×r, where *q* is the total number of time steps.

To align the model outputs with the observed data, the predicted observations $d_{k}^{f}$ (size s×r) are extracted at time steps corresponding to the sensor measurements:

(10) $$d_{k}^{f}=HD_{k}^{f}$$

where *H* is the observation operator, and *s* is the number of observations.

In order to link the biased measurements with the simulated values, the bias equation is applied:

(11) $$d_{j,k}^{f,obs}=a_{j,k-1}^{a}d_{j,k}^{f}+b_{j,k-1}^{a}$$

where $a_{j,k-1}^{a}$ and $b_{j,k-1}^{a}$ are the *j*-th elements of the calibration coefficient vectors $A_{k}$ and $B_{k}$, respectively, obtained after assimilation at iteration k−1. Similarly, $d_{j,k}^{f}$ denotes the *j*-th column of $d_{k}^{f}$, while $d_{j,k}^{f,obs}$ represents the *j*-th column of $d_{k}^{f,obs}$.

The update step is defined as

(12) $$X_{k}^{a}=X_{k-1}^{a}+K_{IES}\left(d^{obs}-d_{k-1}^{f,obs}\right),$$

where $d^{obs}$ is the perturbed observation vector and $d^{m}$ the actual observational data:

(13) $$d^{obs}=d^{m}+\epsilon_{IES}.$$

Here, $\epsilon_{IES}$ is a white noise matrix (size s×r) with variance $\sigma_{obs}^{2}$, and $d^{m}$ is the vector of actual measurements (size s×1). The vector $d^{m}$ is broadcast across all *r* ensemble members to match the dimensions of $\epsilon_{IES}$.

The Kalman gain $K_{IES,k}$ is defined as

(14) $$K_{IES,k}=C_{IES,k}\left(P_{IES,k}+R_{IES,k}+\lambda_{k}{\left(\mathrm{diag}(P_{IES,k})\right)}^{-1}\right),$$

where $R_{IES,k}$ (size s×s) is the observation error covariance matrix, typically diagonal, with diagonal entries representing the variances of individual observations. $P_{IES,k}$ (size s×s) is the observation covariance matrix, approximated as

(15) $$P_{IES,k}\approx \frac{1}{r-1}\sum_{j=1}^{r}\left(d_{j,k}^{f,obs}-{\bar{d}}_{k}^{f,obs}\right){\left(d_{j,k}^{f,obs}-{\bar{d}}_{k}^{f,obs}\right)}^{T},$$

and $C_{IES,k}$ (size m×s) is the cross-covariance matrix, approximated as

(16) $$C_{IES,k}\approx \frac{1}{r-1}\sum_{j=1}^{r}\left(x_{j,k}-{\bar{X}}_{k}\right){\left(d_{j,k}^{f,obs}-{\bar{d}}_{k}^{f,obs}\right)}^{T}.$$

Here, $d_{j}$ (size s×1) is the element vector of *d*, $\lambda_{k}$ is the dynamic stability factor, and $\bar{\left(.\right)}$ denotes the ensemble mean.

### 2.6. Particle Filter

Particle filters (PFs) are a family of data assimilation methods based on ensemble members, widely used and extensively described in the literature. This section provides a general explanation of the method and the specific variant used in this study. In essence, the goal of PFs is to estimate the posterior distribution $p(\theta |d^{\mathrm{obs}})$ of the model parameters θ given the observations $d^{\mathrm{obs}}$. According to Bayes’ theorem:

$$p\left(\theta |d^{\mathrm{obs}}\right)=\frac{p\left(d^{\mathrm{obs}}|\theta \right)\;p\left(\theta \right)}{p(d^{\mathrm{obs}})},$$

where $p(d^{\mathrm{obs}}|\theta )$ is the likelihood of the observations given the parameters, p(θ) is the prior distribution of the parameters and $p(d^{\mathrm{obs}})$ is the marginal likelihood. Since the marginal likelihood $p(d^{\mathrm{obs}})$ is often intractable, particle filters approximate the posterior distribution using a set of particles $\left\{x_{j,k}\right\}$. Sequential Importance Sampling, a key component of PFs, approximates the posterior distribution by iteratively propagating and weighting a set of particles. Each ensemble member $x_{j,k}$ represents a potential realisation of the system’s state and parameters at iteration *k*.

In the prediction step, as similarly described in the previous subsection for the IES, the model propagates each particle independently using the parameter matrix $X_{k-1}^{a}$, producing the forecasted observations $d_{j,k}^{f}$ for all particles. These forecasted observations are then adjusted for measurement biases using the bias error function, resulting in the predicted observations $d_{j,k}^{f,\mathrm{obs}}$.

The update step refines the particle estimates by incorporating the observations into the particle filter. The primary goal is to assign a weight to each particle based on how well its predicted observations agree with the actual observations. This agreement is evaluated using a discrepancy metric, such as the mean squared error (MSE), which quantifies the difference between the predicted and observed values over the entire time horizon length (*s*). The discrepancy is defined as

$${\mathrm{MSE}}_{j,k}=\frac{1}{s}\sum_{t=1}^{s}{\left(d_{t}^{obs}-d_{j,k,t}^{f,obs}\right)}^{2},$$

where $d_{t}^{obs}$ and $d_{j,k,t}^{f,obs}$ represent the actual observation and the predicted observation for particle *j* at iteration *k* and time step *t*. Particles with lower MSE values are assigned higher weights, indicating a closer agreement between their predictions and the observations. These weights are then normalised to approximate the posterior distribution, completing the update step.

To calculate the weights, it is assumed that the observation errors are independent and normally distributed with zero mean and variance $\sigma_{\mathrm{obs}}^{2}$. Therefore, the likelihood of the observations given the particle’s predictions is proportional to

$$p\left(d^{obs}|x_{j,k}\right)\propto \exp\left(-\frac{1}{2\sigma_{\mathrm{obs}}^{2}}\sum_{t=1}^{s}{\left(d_{t}^{obs}-d_{j,k,t}^{f,obs}\right)}^{2}\right)$$

Using this relationship, the weight of each particle is calculated as the negative exponential of the MSE, scaled by the observation variance and the time horizon length *s*:

$$w_{j,k}=\exp\left(-\frac{s}{2\sigma_{\mathrm{obs}}^{2}}\cdot {\mathrm{MSE}}_{j,k}\right).$$

After calculating the weights for each particle, they must be normalised to ensure numerical stability and to make them probabilistically interpretable (i.e., ensuring that they sum to one). Direct normalisation of weights can lead to numerical underflow, particularly when weights are very small due to the exponential function used in their computation. To address this, the log-sum-exp trick is employed, which improves the stability of the normalisation process. It begins by computing the logarithm of the weights, given by

$$\log w_{j,k}=-\frac{s}{2\sigma_{\mathrm{obs}}^{2}}\cdot {\mathrm{MSE}}_{j,k}.$$

Next, the maximum log weight is subtracted from all log weights to prevent numerical underflow:

$$\log{\tilde{w}}_{j,k}=\log w_{j,k}-\max_{j}\left(\log w_{j,k}\right).$$

This ensures that the largest weight is rescaled to zero in the logarithmic space, while all other weights remain proportionally consistent. The rescaled log weights are then converted back to the standard weight space using the exponential function:

$${\tilde{w}}_{j,k}=\exp\left(\log{\tilde{w}}_{j,k}\right).$$

Finally, the weights are normalised by dividing each rescaled weight by the sum of all rescaled weights:

$$w_{j,k}=\frac{{\tilde{w}}_{j,k}}{\sum_{j=1}^{r}{\tilde{w}}_{i,k}}.$$

The final step is resampling, which ensures that computational effort is focused on particles with higher probabilities of representing the true system state. After calculating and normalising the weights, resampling selects particles based on their relative weights. Particles with higher weights are more likely to be retained or duplicated in the updated particle set, while those with lower weights are less likely to be included.

This step prevents weight degeneracy, where only a few particles dominate the representation of the posterior distribution. By redistributing the particles proportionally to their weights, resampling maintains a diverse and efficient set of particles, improving the overall approximation of the posterior. The resampled particle set $X_{k}^{a}$ with equal weights serves as the basis for the next iteration.

### 2.7. Theoretical Basis for Parameter Constraints

The projection method from [22] is employed to ensure that the model parameters remain within physically plausible ranges. In the following, a general overview of the optimisation process used to project the parameters onto the feasible set defined by the constraints is provided. For a more detailed explanation of the method, please refer to [22].

The projection of each ensemble member or particle $x_{j,k}^{a}$ from the updated parameter matrix $X_{k}^{a}$ onto the feasible set defined by the constraints involves solving an optimisation problem. This minimises the Mahalanobis distance between the unconstrained update $x_{j,k}^{a}$ and the closest point in the constrained space, weighted by the inverse of the updated parameters’ covariance matrix $C_{X^{a}}$, while satisfying the constraints. This can be formulated as

(17) $$x_{j,k}^{a,c}=argmin_{x}\frac{1}{2}{∥x-x_{j,k}^{a}∥}_{C_{X^{a}}^{-1}}^{2}\;s.t.\;c^{a}\left(x\right)\leq 0$$

where $c^{a}$ is the vector of constraints and may contain nonlinear or linear inequalities and equalities.

The iterative solution of Equation (17) is achieved by linearising the constraints and iteratively updating $x_{j,k}^{a,c}$ until all the constraints are fully satisfied. Linearisation simplifies the non-linear constraints into a form that can be iteratively solved using the Jacobian matrix. The minimisation problem, when linearised, is defined as

(18) $$x_{j,k}^{a,c}=argmin_{x}\frac{1}{2}{∥x-x_{j,k}^{a}∥}_{C_{X^{a}}^{-1}}^{2}\;s.t.\;Jx=R,$$

where *J* is the transpose of the Jacobian matrix of the constraints, $J=\left(\nabla_{x}{\left(c^{a}\right)}^{T}\right){|}_{x=x_{j,k}^{a}}$, and $R=Jx_{j,k}^{a}-c^{a}{|}_{x=x_{j,k}^{a}}$.

To derive the update equation, we set the derivative of the Lagrangian for the linearised constraint sub-problem to zero, as shown here:

(19) $$L\left(x,\lambda \right)=\frac{1}{2}{∥x-x_{i,k}^{a}∥}_{C_{X^{a}}^{-1}}^{2}+{\left(R-Jx\right)}^{T}\lambda ,$$

The Lagrange multipliers can be determined by solving $\nabla_{x}L=0$ and substituting *x* into the solution of $\nabla_{\lambda }L=0$, resulting in $\lambda ={\left(JC_{X^{a}}J^{T}\right)}^{-1}\left(R-Jx_{i,k}^{a}\right)$.

Then, the constrained updates are calculated by substituting λ back into the solution of $\nabla_{x}L=0$, and is given by

(20) $$x_{j,k}^{a,c}=x_{j,k}^{a}+C_{X^{a}}J^{T}{\left(JC_{X^{a}}J^{T}\right)}^{-1}\left(R-Jx_{i,k}^{a}\right)$$

This equation provides the update for the parameter vector that simultaneously minimises the distance to the unconstrained solution while satisfying the constraints.

The projection method from [22], originally applied to ensemble members in an IES, is adapted here for particles in a PF. In this framework, particle states are updated based on the likelihood from observations, and their parameters are projected onto the feasible set to ensure they remain physically plausible. This projection minimises the Mahalanobis distance between the unconstrained and constrained parameters, following the same optimisation approach as in the IES but applied to individual particles.

### 2.8. Convergence Criterion

The stopping criterion employed in the data assimilation algorithms is based on monitoring the relative change in parameter estimates across iterations. For each iteration *k*, the relative change in the current parameter means with respect to the initial parameter means is calculated. Then, the mean absolute change between the current parameter means and those from the previous iteration is determined.

If this mean change is smaller than a predefined threshold ϵ, the parameters are considered to have converged, and the assimilation process is stopped. This approach ensures that the algorithm continues adjusting the parameters only while significant changes occur, avoiding unnecessary iterations once the parameters have stabilised.

The algorithm stops when the average normalised change in parameter estimates falls below the threshold ϵ:

$$\delta_{k}=\frac{1}{m}\sum_{i=1}^{m}\left|\frac{\mu_{k,i}-\mu_{k-1,i}}{\mu_{0,i}}\right|,$$

where:

- *m*: Total number of parameters (as previously defined in Section 2.4).
- $\mu_{k,i}$: Mean of parameter *i* in the updated parameter matrix $X_{k}^{a,c}$ at iteration *k*.
- $\mu_{k-1,i}$: Mean of parameter *i* in $X_{k-1}^{a,c}$ at iteration k−1.
- $\mu_{0,i}$: Initial mean of parameter *i* in $X_{0}$.

If $\delta_{k}<\epsilon$, the algorithm has converged.

## 3. Results

This section presents the results of the data assimilation algorithms used to calibrate soil moisture sensors. Initially, a synthetic case was employed to evaluate the capabilities and limitations of the proposed methods under controlled conditions. This preliminary approach was essential for understanding the behaviour of the algorithms under idealised conditions. In both the synthetic and real cases, various configurations were explored, including different ensemble sizes and observation error levels. These configurations were critical for testing the methods’ performance under varying conditions and for fine-tuning the algorithms for optimal results in each scenario.

Subsequently, the methods were applied in a real-world scenario using data from a farm. This scenario involved a complex and dynamic environment, where variations in environmental and soil conditions posed additional challenges to data assimilation. The transition from the synthetic case to real-world application validated the algorithms’ effectiveness in operational settings, highlighting their potential to improve the accuracy of low-cost sensors for automated irrigation systems.

### 3.1. Synthetic Case

The performance of the proposed data assimilation methods was evaluated using a synthetic soil moisture time series generated with the Hydrus 1D software. This synthetic dataset provides a controlled environment where the true soil moisture dynamics are known, allowing for assessment of the calibration and parameter estimation procedures.

#### 3.1.1. Flow Domain and Boundary Conditions

The simulation was performed on a one-dimensional soil profile with a depth of 50 cm, discretised into 50 nodes to capture soil moisture dynamics. The soil was characterised as silty loam, a common agricultural soil type. Although the entire soil profile was modelled, only the data from a single point located 10 cm below the soil surface were utilised to simulate the readings of one sensor.

The lower boundary condition was set to free drainage, allowing water to exit the system unimpeded, simulating natural gravitational drainage. The upper boundary condition was defined as an atmospheric boundary condition with a surface layer, incorporating water input through irrigation events.

#### 3.1.2. Irrigation Events

To replicate real-world agricultural practices, irrigation events were incorporated into the simulation. Irrigation was applied once per day for a duration of one hour, following a periodic schedule over the 80 h simulation period. During irrigation events, the irrigation flux magnitude $k_{u}$ was set to 0.005 cm/min.

#### 3.1.3. Parameter Settings

The true soil moisture time series, representing the VWC at 10 cm, was generated using the van Genuchten parameters representative of silty loam soil, as shown in Table 1. These parameters define the soil hydraulic properties and were used to generate the reference or true soil moisture time series.

The initial condition for the soil profile was set to a uniform VWC of $\theta_{0}=0.35$. Before assimilating observations, a model warm-up period was implemented to minimise the influence of uncertainty in the initial conditions. During this warm-up, the model was run without assimilation until the arbitrary initial condition was effectively “forgotten”, allowing the model to reach a dynamic equilibrium determined by its physics and boundary conditions [25].

#### 3.1.4. Description of Test Cases

To assess the impact of ensemble size (number of ensemble members or particles) on the performance of the data assimilation methods, three different values of *r* were evaluated: r=25, r=50 and r=100. Each ensemble member represents a potential realisation of the parameter set. Varying *r* allows us to examine how the number of ensemble members affects the accuracy and stability of the parameter estimates in both IES and PF methods.

#### 3.1.5. Implementation Details

The data assimilation methods were applied following the methodology described in Section 2. The initial parameter matrix $X_{0}$ was initialised by sampling from prior distributions, reflecting reasonable uncertainty based on typical soil properties and sensor characteristics. The initial assumptions and the implementation of the model warm-up period were crucial for reducing the influence of arbitrary initial conditions and ensuring that the assimilation focused on correcting model parameters rather than compensating for incorrect initial states.

Table 2 summarises the mean and variance used in the prior parameter distributions:

To incorporate drift error into the VWC time series and simulate sensor drift, the values $a_{\mathrm{true}}=1.15$ and $b_{\mathrm{true}}=0.07$ were used according to Equation (6). These parameters serve as calibration coefficients to adjust the model-generated time series, mimicking the behaviour of a sensor affected by drift. Gaussian noise was omitted from the synthetic data to create a controlled environment for validating the assimilation methods and assessing their intrinsic performance. This approach ensures that any estimation errors are exclusively due to the methods themselves.

The data assimilation process involved iteratively updating the parameter estimates by integrating the synthetic observed data with the numerical model, as detailed in Section 2. For each ensemble size *r*, the methods were executed until convergence was reached based on the criterion specified in Section 2.8. The final estimated parameters were obtained from the converged parameter matrix $X_{k}^{a}$.

#### 3.1.6. Results of the Synthetic Case

Table 3 presents the estimated parameters obtained from both the IES and PF methods for each ensemble size, alongside the true parameter values used to generate the synthetic data. For both methods, increasing *r* seems to lead to a slight improvement in prediction accuracy for some parameters when compared to the true values. For the IES method, the error reduction is particularly notable for parameters such as *a* and *b*. For example, for *a*, the prediction improves from 1.336 with *r* = 25 to 1.156 with *r* = 100, approaching the true value of 1.15. In contrast, the PF method does not exhibit a clear trend of increasing accuracy with larger sample sizes across all parameters. With the exception of α, the improvement in accuracy as *r* increases is inconsistent or marginal for most parameters. For example, the prediction for α improves significantly from 0.0107 with *r* = 25 to 0.0022 with *r* = 100, indicating a benefit from larger sample sizes. This lack of clear improvement suggests that the PF method may not be as robust in handling the increased number of samples for all parameters, potentially due to its sensitivity to particle resampling and the complex interactions between parameters in the model.

**Figure 2:**
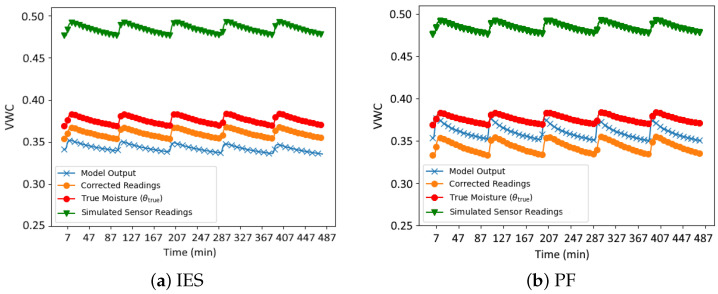
Time series comparison of synthetic soil moisture data assimilation using the IES (**a**) and PF (**b**) methods.

Figure 2 illustrates a comparison of soil moisture time series as an example for the case in which r=100 ensemble members were used. The figure contrasts the corrected sensor readings $\theta_{c}$ (adjusted using the $a^{a}$ and $b^{a}$ coefficients obtained after data assimilation) with the true soil moisture values ($\theta_{true}$), the simulated sensor readings, which serve as input for the data assimilation methods and the Hydrus model output, generated using the hydraulic parameters estimated by the assimilation methods.

It can be seen how, for both methods, the corrected readings (orange) align more closely with the true moisture values, althoug some differences between methods can be identified. In the case of IES (Figure 2a), the corrected readings demonstrate a better alignment with the true soil moisture over the entire time series. Conversely, the PF method (Figure 2b) shows corrected readings that, while improved relative to the uncorrected sensor data, still exhibit some discrepancies.

Table 4 provides the root mean square error (RMSE) values between the true soil moisture series and the corrected series for different ensemble sizes (r=100, r=50, and r=25). The IES method consistently outperforms PFs in terms of error reduction, as evidenced by the lower RMSE values across all sample sizes. For r=100, IES achieves an RMSE of 0.0198, which is significantly lower than the PF value of 0.0313. This trend persists as the sample size decreases, with IES maintaining relatively stable RMSE values, while the PF method exhibits a noticeable increase in error, particularly r=25 (RMSE = 0.0388).

### 3.2. Application to Field Data

This section summarises the results of an experimental field validation of the proposed algorithms at an on-production farm in Dos Hermanas (Spain). The farm features a drip irrigation system and the soil at the site is classified as silty loam, a texture that combines moderate levels of sand, silt, and clay, making it ideal for agricultural activities due to its good water retention and drainage properties. This specific soil type was considered to refine the initial estimations of soil parameters within the model.

#### 3.2.1. Sensors Employed and Initial Calibration

Data acquisition was conducted using SoilWatch 10 sensors (Pino-Tech, Strachocin, Poland), which employ capacitive technology to measure the soil’s water content. This low-cost sensor, priced at approximately 20, is an affordable option for widespread use in agricultural and environmental monitoring [26]. Prior to deployment, a calibration process was conducted using soil samples collected from the farm. This calibration was necessary to convert the sensor’s voltage output to VWC. The process involved correlating the sensor’s voltage readings with gravimetric measurements of soil moisture content, resulting in a quadratic relationship between the sensor’s output and the actual water content in the soil, as detailed in [27]. Regardless of the initial calibration’s precision, the data assimilation process is designed to iteratively improve the estimates. As demonstrated later by the validation data, even though the initial calibration lacked high precision, the data assimilation method effectively corrected for measurement biases through its bias-aware approach, ensuring accurate soil moisture estimations. In the deployment, the sensor was installed at a depth of 10 cm below the soil surface. The dataset for assimilation was collected over approximately five days, from 12 to 17 April 2024.

For validation purposes, the ThetaProbe ML3 sensor (Delta-T Devices Ltd., Cambridge, UK) was employed. The ThetaProbe is widely recognised for its high precision and reliability, making it a standard in soil moisture measurement. Unlike the SoilWatch 10, the ThetaProbe utilises VWC measurements by employing a frequency-domain reflectometry (FDR) technique, providing robust data for comparison. The ThetaProbe was positioned adjacent to the SoilWatch 10 sensor, inserted into the soil as close as possible without disturbing the surrounding soil or affecting the SoilWatch 10 measurements. Prior to use, the ThetaProbe was calibrated for the farm’s soil type following the manufacturer’s guidelines [28]. Calibration involved correlating the sensor’s voltage readings with gravimetric soil moisture measurements. Soil samples were collected, weighed and their sensor voltage recorded. After drying at 105 °C, the dry weights and corresponding voltages were measured. These data were used to calculate ThetaProbe calibration coefficients $a_{0}$ and $a_{1}$, which were input into the HH2 Moisture Meter to provide VWC readings. Although the buried sensors limited the ability to take multiple measurements around the sampling point, measurements were taken close to the SoilWatch 10 sensor to reduce variability, assuming negligible variation in soil properties over small distances. This minimised the influence of soil heterogeneity on moisture readings and ensured accurate comparisons between sensors. The high accuracy of the ThetaProbe (±1% VWC in mineral soils after calibration [28]) ensured reliable reference measurements.

#### 3.2.2. Model Setup

For the field data assimilation, the same soil profile and boundary conditions as in the synthetic case were used to maintain consistency and comparability. The soil profile was modeled as a one-dimensional domain with a depth of 50 cm, discretised into 50 nodes. The lower boundary condition was set to free drainage, and the upper boundary condition was specified as an atmospheric boundary condition with a surface layer.

Irrigation events were modelled using the irrigation flux magnitude $K_{u}$, which defines the intensity of water applied during irrigation, based on the observed schedule at the farm. The vector *U* was introduced to explicitly represent the timing of irrigation events. This vector assigns a value of 1 at timestamps when irrigation occurred and 0 otherwise.

#### 3.2.3. Initial Parameters and Constraints

The initial parameter matrix $X_{0}$ was initialised by sampling from prior distributions of the parameters, reflecting reasonable uncertainty based on typical soil properties and sensor characteristics for silty loam soils. The means and variances used are shown in Table 5.

The value of the saturated water content $\theta_{s}$ was previously measured in a laboratory test using the oven-drying method, and the value obtained was 0.496. Therefore, $\theta_{s}$ was considered known and did not take part in the assimilation process.

In the selection of the constraints for the van Genuchten parameters, this study focused on parameters proposed in [29] for textural classes close to silty loam according to the soil texture triangle. These classes include Silt, Silty Clay Loam, Loam, Sandy Loam and Clay Loam. The maximum and minimum values for each parameter were determined by the highest and lowest values found among these surrounding textural classes. Table 6 provides the maximum and minimum values for the van Genuchten parameters.

An additional restriction was applied to ensure that the corrected sensor readings did not surpass the predicted saturation moisture content of the soil. This condition maintains the physical consistency of the measurements, as the sensor readings should not indicate a moisture level higher than what the soil can theoretically hold at saturation. The restriction was implemented through the following inequality:

(21) $$\max\left(\mathrm{sensor}\;\mathrm{reading}\right)\leq \theta_{s,i,k}^{a}\cdot a_{i,k}^{a}+b_{i,k}^{a}$$

where max(sensorreading) represents the maximum value of the sensor readings, and $a_{i,k}^{a}$ and $b_{i,k}^{a}$ are the estimated calibration coefficients at iteration *k*.

#### 3.2.4. Evaluation Cases

The impact of ensemble size (number of ensemble members or particles) and observational error variance on the performance was evaluated. Three different ensemble sizes were used: r=25, r=50 and r=100. Additionally, three different standard deviations for observation errors were used: $\sigma_{\mathrm{obs}}=0.01$, $\sigma_{\mathrm{obs}}=0.02$ and $\sigma_{\mathrm{obs}}=0.03$.

The data assimilation process involved iteratively updating the parameter estimates by integrating the observed SoilWatch 10 sensor data with the numerical model, as detailed in Section 2. For each combination of *r* and $\sigma_{\mathrm{obs}}$, the methods were executed until convergence was achieved according to the criterion specified in Section 2.8. The final estimated parameters, including the calibration coefficients *a* and *b*, were obtained from the converged parameter matrix $X_{k}^{a}$.

#### 3.2.5. Validation

The validation dataset was collected using the ThetaProbe ML3 sensor over a slightly extended period, starting on 25 April 2024, eight days after the end of the assimilation period, and lasting just over one day. Unlike the SoilWatch 10 sensor, whose measurements were recorded automatically every 15 min, the ThetaProbe measurements were taken manually using the readout unit and therefore were not collected at regular intervals. No measurements were taken during the night of April 25th and the early morning of April 26th. In total, 49 measurements were taken during this period.

To align the ThetaProbe data with the SoilWatch 10 data for comparison, linear interpolation was applied to the ThetaProbe measurements to fill the gaps and generate a time series corresponding to the SoilWatch 10 timestamps. During the validation period, the magnitude of the irrigation parameter changed due to modifications in the irrigation system. However, this change is not critical, as the primary objective of the validation was to verify the correction of the SoilWatch 10 sensor readings using the estimated calibration coefficients $a^{a}$ and $b^{a}$, rather than to validate the model parameters. The corrected SoilWatch 10 readings were obtained by applying Equation (7). The performance of the data assimilation methods was evaluated by comparing the corrected SoilWatch 10 measurements $\theta_{\mathrm{est}}$ with the ThetaProbe measurements, using the RMSE as the evaluation metric.

#### 3.2.6. Results of the Field Application Case

**Figure 3:**
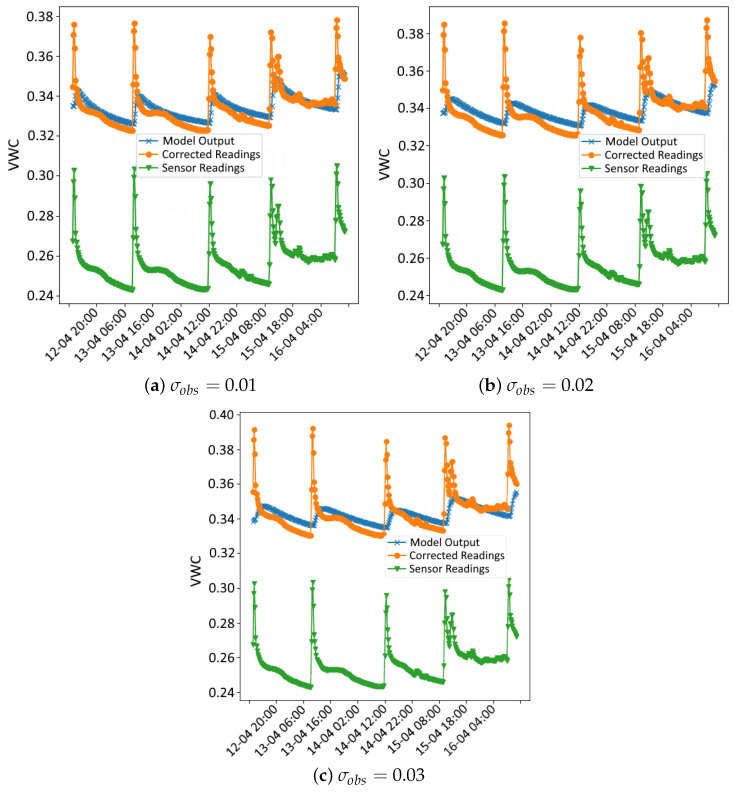
Assimilation results using the IES method for different levels of observation noise ($\sigma_{\mathrm{obs}}$). The plots compare model output (blue), corrected sensor readings $\theta_{c}$ (orange), and original sensor readings (green) for each noise level.

**Figure 4:**
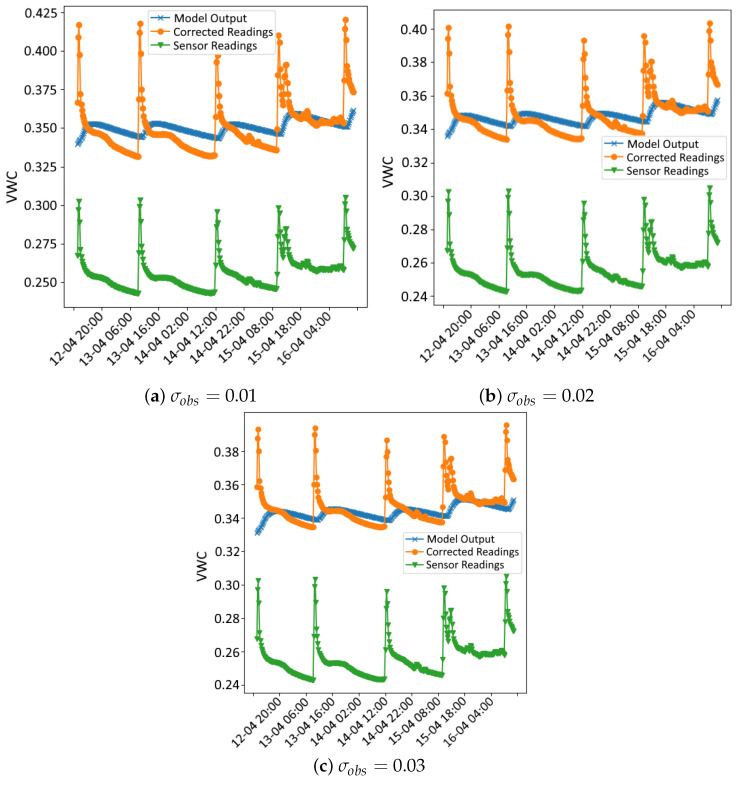
Assimilation results using the PF method for different levels of observation noise ($\sigma_{\mathrm{obs}}$). The plots compare model output (blue), corrected sensor readings $\theta_{c}$ (orange) and original sensor readings (green) for each noise level.

The outcomes of the data assimilation process are illustrated in a series of figures. Figure 3 and Figure 4 illustrate the data used in the assimilation process for both methods for $\sigma_{obs}$ values of 0.01, 0.02 and 0.03, respectively, and *r* = 100, as an example. In each figure, three time series are plotted: the sensor readings (green), the corrected readings ($\theta_{c}$) using the calibration coefficients from the data assimilation process (orange) and the VWC time series simulated by the Hydrus model using the hydraulic parameters obtained in the data assimilation process (blue).

In all cases, even with different values of *r*, notable peaks in the sensor data occur during irrigation events, reflecting the immediate and rapid increase in soil moisture from the drip irrigation system. While the Hydrus 1D simulation does not fully capture the sharp peaks following these events, it shows good alignment with the corrected sensor data during the intervals between irrigations, particularly after the water redistributes and moisture levels stabilise. This indicates that the model effectively simulates the longer-term moisture trends after irrigation events, even if it misses some of the immediate dynamics.

One of the possible reasons for the mismatch during irrigation events stems from the limitation of the Hydrus 1D model, which only simulates vertical water movement and does not account for lateral water spread, which is significant in drip irrigation systems. In drip irrigation, water disperses both vertically and laterally, creating a wetting bulb around the emitter. This localised water distribution leads to rapid increases in soil moisture near the emitter, which are quickly detected by the nearby sensors. These sensors are highly sensitive to localised changes in soil moisture, particularly those near the surface, and are able to capture the sharp peaks right after irrigation. However, because Hydrus 1D cannot simulate lateral water movement, it smooths out these localised increases, resulting in a more gradual moisture curve that misses the sharp spikes.

Despite these limitations, the Hydrus 1D model shows good performance in simulating the longer-term moisture behaviour, particularly after the effects of the irrigation peaks dissipate. The corrected sensor readings align well with the Hydrus 1D simulation once the water has redistributed deeper into the soil and the short-term effects of irrigation have subsided. This alignment shows that Hydrus 1D is effective in capturing the general water infiltration and redistribution trends.

As for the validation analysis, Table 7 shows the validation results for the IES and PF methods across different values of *r* (25, 50 and 100) and $\sigma_{obs}$ (0.01, 0.02 and 0.03). For the IES method, the results show minimal variation in corrected RMSE as the *r* value increases. For instance, at an $\sigma_{obs}$ of 0.01, the RMSE slightly changes from 0.0483 (ensemble size 25) to 0.0533 (ensemble size 100). However, the corrected RMSE shows a slight decrease as the $\sigma_{obs}$ value increases, with the best result being 0.0384 for an *r* value of 50 and an observational error of 0.03. In contrast, the PF method exhibits a more pronounced improvement in performance, particularly as the $\sigma_{obs}$ increases. At an *r* of 100, the PF achieves a significantly lower RMSE of 0.0309 ($\sigma_{obs}$ = 0.01) compared to the IES method for the same value, which further reduces to 0.0164 for an $\sigma_{obs}$ of 0.03, this being the best result achieved.

Both the IES and the PF methods lead to a significant improvement in accuracy compared to the uncorrected sensor, which has an RMSE of 0.108. For the IES method, the lowest corrected RMSE achieved is 0.0384, representing a 64.4% improvement in accuracy. Similarly, the PF, which demonstrates the best performance with an RMSE of 0.0164, achieves an even more substantial improvement of 84.8%. These results highlight that, while both methods considerably enhance the sensor’s precision, the PF outperforms the IES method, particularly at higher observational errors and smaller values of *r*.

The performance of the IES and PF methods varies depending on the characteristics of the data. With synthetic data, the IES demonstrated superior performance compared to the PF. This advantage is likely due to the synthetic data’s linear bias and absence of random noise, which align well with the IES’s assumptions of linearity and Gaussian error. These conditions enable the IES to efficiently converge towards the true model and bias parameters.

However, the situation changes with real-world data, which often include non-Gaussian noise, non-linearity, and un-modelled discrepancies such as soil heterogeneity, temporal variability and spatial variability due to drip irrigation patterns. Under these conditions, the PF method outperforms the IES. Unlike the IES, the PF method does not rely on linear approximations or Gaussian assumptions. Instead, it employs weighted particles to represent the posterior distribution, allowing it to capture complex, non-linear relationships and adapt to non-Gaussian noise. This flexibility makes the PF method more robust against model discrepancies and uncertainties, resulting in better agreement with validation data.

The superior performance of the PF with real-world data can be attributed to its ability to handle the inherent complexities of field observations, such as non-linearity and variability in irrigation patterns. Conversely, the IES’s reliance on its assumptions limits its effectiveness in these scenarios. These differences highlight how the characteristics of the data influence the comparative performance of the two methods. While the IES excels in synthetic scenarios where its assumptions hold, the PF adaptability gives it an advantage in the challenging context of real-world soil moisture measurements.

**Figure 5:**
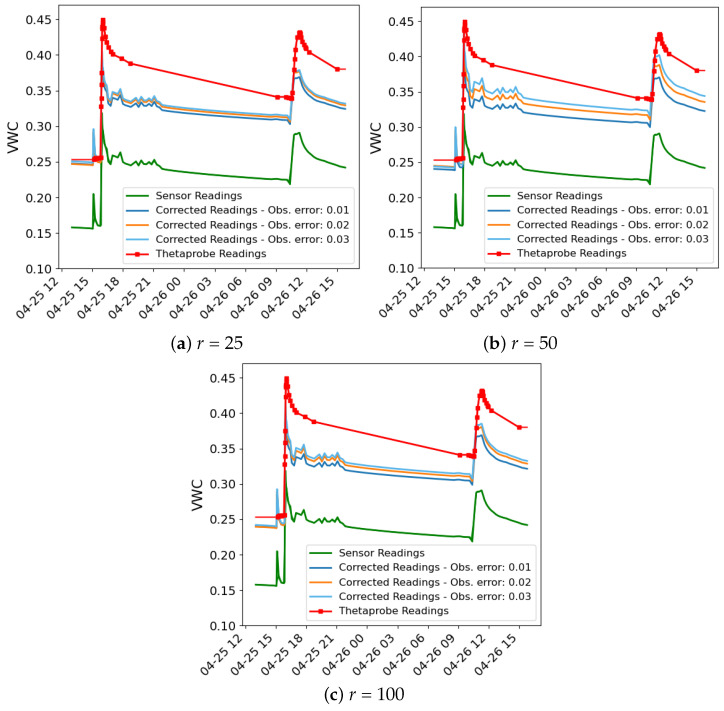
VWC time series of the sensor readings (green), the ThetaProbe sensor readings (red) and the corrected readings for the IES method at r=100 with varying values of $\sigma_{\mathrm{obs}}$ (blue, orange and light blue).

**Figure 6:**
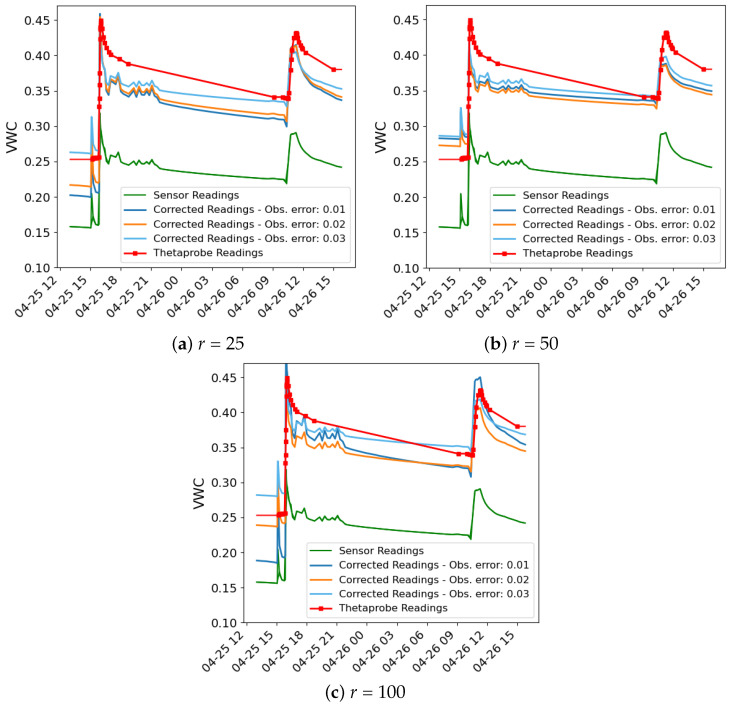
VWC time series of the sensor readings (green), the ThetaProbe sensor readings (red) and the corrected readings for the PF method at r=100 with varying values of $\sigma_{\mathrm{obs}}$ (blue, orange and light blue).

Figure 5 and Figure 6 show the time series of moisture from the sensor readings, the ThetaProbe sensor readings and the corrected readings for both methods, with different values of *r* and $\sigma_{obs}$. As can be observed, the sensor readings show a significant bias compared to those of the ThetaProbe. In all cases, the corrected readings are closer to the ThetaProbe readings.

Table 8 and Table 9 present the predicted soil parameter values using the IES and the PF methods, respectively, for an ensemble size of 100 and $\sigma_{obs}$ values of 0.01, 0.02 and 0.03, with initial parameter values provided for comparison. Although the true values of the soil parameters are not known, the predicted results across both methods do not deviate significantly from the initial values, which are consistent with the expected ranges for a silty loam soil.

For the IES method (Table 8), the predicted values show slight adjustments from the initial parameters. The parameter $\theta_{r}$ remains close to the initial value (0.067), fluctuating between 0.0647 and 0.0678 across the different noise levels. The other soil parameters, such as α, *n* and $k_{s}$, also exhibit relatively small changes, with α ranging from 0.0012 to 0.0063 and *n* showing a small upward trend as the noise level increases.

The Particle Filter method (Table 9) shows similar trends, with slight variations in the predicted values. For example, the parameter $\theta_{r}$ varies from 0.0659 to 0.0739, while α and *n* show minimal changes from the initial values. Interestingly, $k_{s}$ and $k_{u}$ are slightly more variable in the PF results compared to IES, but still remain within a reasonable range for silty loam soils.

Table 10 shows that there is no significant difference in execution time between the IES and PF methods, especially for smaller ensemble sizes. For an ensemble size of 25, both methods have similar runtimes (22 min for IES and 19 min for PF). Although the execution time increases with ensemble size, the difference remains modest (84 min for IES and 104 min for PF at size 100). The primary delay in both methods is attributed to the Hydrus 1D software employed for prediction calculations, rather than the convergence time of the algorithms themselves.

Although the data assimilation algorithms require significant execution times, especially for larger ensembles, real-time correction is unnecessary in this context. Soil moisture sensor drift occurs gradually over long periods of time [30], meaning that, once corrected, sensors maintain improved accuracy for an extended period. Additionally, the methods provide updated calibration coefficients that can be used to adjust future sensor readings without reapplying the computational algorithms. Thus, these techniques function as an efficient offline calibration tool rather than a real-time correction mechanism.

## 4. Concluding Remarks: Sensor Calibration in Irrigation Management

This section evaluates the impact of sensor calibration on the performance of irrigation systems under simulated conditions, focusing on its potential economic and environmental benefits. The analysis centers on water savings, cost reduction, minimising water pump start-ups and preventing plant water stress due to insufficient irrigation. A Model Predictive Control (MPC) system is employed to simulate hypothetical scenarios wherein the irrigation control system depends on sensors with varying levels of bias. This advanced control strategy, widely used in industrial and process control, is assessed to understand how the proposed calibration methods improve irrigation efficiency by correcting sensor biases.

MPC continuously predicts the system’s future behaviour over a defined time horizon, relying on a dynamic process model. It then optimises the control inputs by solving an optimisation problem at each control step, based on a set of constraints and an objective function that typically aims to minimise error, resource consumption or operational costs. This approach allows MPC to handle multivariable systems with constraints on control actions and system states, making it particularly effective in complex environments like irrigation, where various factors such as soil moisture, weather forecasts and water availability must be considered [31].

The simulation models were derived from real data collected at the Dos Hermanas farm, as mentioned in the previous section. Both non-linear and linear models were obtained. For the plant model, the non-linear model of one of the sensor nodes was utilised, while the linear model of the same node was used as the MPC model. The details of the implementation, data acquisition and model development can be found in the following work [32].

The simulation followed the methodology in [33], maintaining the same parameters and constraints but employing a conventional MPC case instead of an adaptive one for simplicity. The difference lay in the introduction of errors due to the miscalibration of the *b* parameter, which affected the plant output readings (specifically, the moisture value used by the controller). Errors in the *b* parameter were introduced, and their impact on water consumption and pump start-ups was evaluated. This metric is critical, as energy consumption and pump lifespan are directly influenced by these factors. The simulation was conducted over a period of four days, with maximum and minimum moisture limits set at 0.39 and 0.16, respectively. These limits defined the range within which the MPC was required to maintain soil moisture levels.

The selected values were determined in consultation with an agronomist responsible for irrigation management on the farm, whose expertise informed the implementation process. Notably, the controller operates without relying on predefined reference values. Instead, the optimisation function incorporates upper and lower constraints on soil moisture levels, as well as cost considerations for pump operation, state changes (to reduce startup frequency and wear) and irrigation schedules adjusted for weather conditions.

The scenarios evaluated are the following:

1. Overestimated measurements (*b* = 0.12): In this case, the sensor readings are overestimated, resulting in insufficient irrigation. This scenario evaluates the water stress on plants caused by inadequate irrigation. The parameter b=0.12 was chosen to reflect a similar reading error observed in the field validation from the previous section.
2. Underestimated measurements (*b* = −0.12): In this scenario, the sensor readings are underestimated, leading to excessive irrigation. It assesses the impact of over-irrigation on both water usage and associated costs. The value b=−0.12 was again selected to represent a comparable error as in the field validation case.
3. Corrected measurements (*b* = 0.012 and *b* = −0.012): This case assumes a best-case calibration scenario where the PF corrects up to 90%, similar to the correction observed in the validation case discussed previously. Both underestimated and overestimated readings are considered here, and the effect of the calibration method is evaluated.
4. Optimal case (*b* = 0): In this case, it is assumed that the sensor measurements are fully accurate. The MPC would therefore provide an optimal irrigation strategy.

Table 11 presents the results for the scenarios. It can be observed that, in the case where b=−0.12, water consumption exceeds the optimal case by 2520 L, and the number of pump start-ups is more than doubled. Conversely, in the case where b=0.12, 1060 L less water is used, which could lead to water stress for the plants as it falls below the optimal level (b=0). In the following cases, with b=0.012 and b=−0.012, it is observed that, for the first case, both water consumption and the number of pump start-ups are the same as in the optimal case. For the second case, there is only a small difference of 120 L in water consumption, while the number of pump start-ups remains the same.

**Figure 7:**
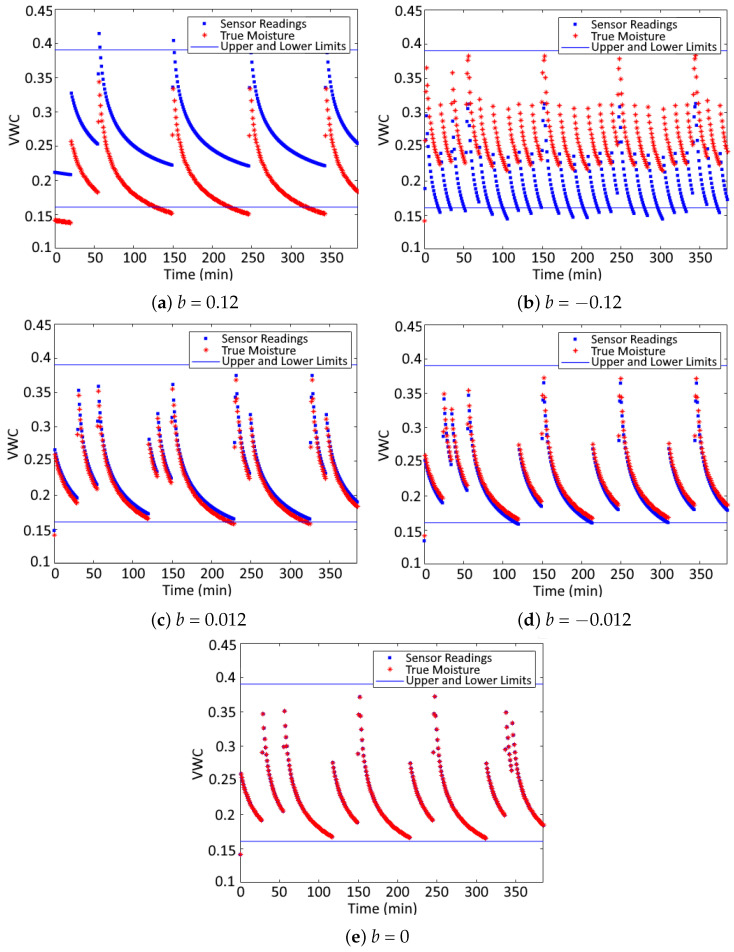
Moisture time series for each analysed scenario.

In Figure 7, the time series of both the true moisture (*b* = 0) and the simulated sensor readings for the different cases analysed are shown, as well as the minimum and maximum moisture limits. In Figure 7a, it is observed that the moisture level falls below the minimum threshold during significant periods, potentially causing water stress in the crops. In Figure 7b, an excessive number of moisture peaks related to motor start-ups can be seen. It is also observed that the moisture levels remain relatively higher for longer periods. In Figure 7c,d, the cases with corrected measurements are shown, where the irrigation patterns and strategy are quite similar to the optimal case in Figure 7e.

In conclusion, the findings highlight the substantial impact of sensor calibration on irrigation system performance. Miscalibration of the *b* parameter, as observed in both overestimated and underestimated scenarios, leads to sub-optimal irrigation, either causing water stress to the plants or excessive water consumption and increased wear on the pump. Notably, the calibration scenarios corrected by up to 90%—a precision level that can be achieved through data assimilation, as demonstrated in the field application using the PF method—closely matched the optimal case. This underscores the critical role of data assimilation in enhancing sensor accuracy and optimising irrigation control. The results highlight the crucial role of precise sensor calibration, which can be achieved swiftly and practically through data assimilation, thereby securing both economic and environmental benefits, such as water conservation, energy savings, reduced operational costs and extended lifespan.

## 5. Conclusions

This study introduces a low-cost soil moisture sensor calibration approach using data assimilation, specifically applied to a drip irrigation system. The method integrates the Hydrus 1D model with PF and IES techniques to enhance sensor reading accuracy, thereby optimising water use in automated irrigation systems.

The data assimilation approach was validated in both synthetic and real agricultural environments, resulting in significant improvements in sensor accuracy, particularly with the PF method, which achieved an 84.8% improvement compared to the original readings. This improvement was achieved by correcting sensor biases, as data assimilation identified correction parameters. In the field, the corrected readings aligned well with the reference measurements from the ThetaProbe sensor, demonstrating the effectiveness of the approach for real irrigation systems.

Additionally, physical constraints were applied on the model parameters to ensure that the adjusted values remained within physically plausible limits, maintaining consistency with soil properties and water flow processes. These constraints were important to ensure the model’s validity and applicability in real field conditions, providing a robust and practical solution for improving the efficiency of precision irrigation systems.

The concluding remarks section addressed the impact of sensor calibration on irrigation system performance, particularly regarding water savings, cost reduction and reduced pump wear. In the tested case using a predictive control system, accurate calibration saved up to 2400 L of water over four days and significantly reduced unnecessary pump activations. Scenarios with overestimated or underestimated moisture measurements resulted in inefficient water usage. However, calibration corrections achieved an optimal balance in water consumption and system performance.

## 6. Further Works

One challenge in this study was accurately modelling the spatial flow of water in a drip irrigation system, as evidenced by discrepancies between the model’s predicted parameters and real data. Future studies will address this issue by enhancing model quality using the 2D version of Hydrus, enabling more accurate water flow modelling and improved data assimilation outcomes. Expanding the research to larger farms with distributed sensor networks will offer insights into the scalability of the proposed calibration method in large-scale agricultural applications. This research will involve real-time calibration across extensive sensor networks, further enhancing water management efficiency at scale.

Future research will explore integrating deep learning models to enhance the calibration process’s adaptability. Leveraging historical sensor and environmental data, machine learning can provide a continuous and highly accurate calibration framework, further optimising system performance in dynamic agricultural settings. Additionally, testing the methodology across diverse soil types and crop conditions is essential to ensure its robustness and generalisability.

## Figures and Tables

**Table 1:** Reference values of hydraulic and irrigation parameters.

Parameter	$\theta_{r}$	$\theta_{s}$	α(1/cm)	*n*	$k_{s}\;(\mathrm{cm}/\min)$	$k_{u}\;\left(\mathrm{cm}/\min\right)$
Reference value	0.067	0.45	0.02	1.41	0.0075	0.005

**Table 2:** Mean and variance used in the prior parameter distributions.

Parameter	$\theta_{r}$	α	*n*	$k_{s}$	$k_{u}$	*a*	*b*
Mean	0.05	0.01	1.35	0.01	0.002	1.0	0.0
Variance	$10^{-4}$	$2.25\times 10^{-4}$	0.02	$10^{-4}$	$3.6\times 10^{-5}$	0.01	0.03

**Table 3:** Estimates of the Van Genuchten model parameters, $k_{u}$, and calibration parameters using the IES and PF methods with *r* values of 25, 50 and 100 for the application case with synthetic data.

Parameter	r	$\theta_{r}$	α	*n*	$k_{s}$	$k_{u}$	*a*	*b*
True value		0.067	0.02	1.41	0.0075	0.005	1.15	0.07
IES estimates	25	0.0541	0.0212	1.3542	0.0169	0.00412	1.336	0.007
	50	0.0486	0.0195	1.4197	0.0100	0.00465	1.187	0.061
	100	0.0506	0.0194	1.3933	0.0124	0.0046	1.156	0.067
PF estimates	25	0.0523	0.0107	1.3129	0.0060	0.0018	1.161	0.115
	50	0.0538	0.0132	1.7382	0.0061	0.0034	1.033	0.124
	100	0.0416	0.0022	1.6034	0.0052	0.0014	1.030	0.133

**Table 4:** The RMSE values of the true moisture series versus the corrected moisture series were calculated for each method and for different values of *r*.

r	100	50	25
IES	0.0198	0.0187	0.0195
PF	0.0313	0.0308	0.0388

**Table 5:** Mean and variance used in the prior parameter distributions for the field application.

Parameter	$\theta_{r}$	α	*n*	$K_{s}$	$K_{u}$	*a*	*b*
Mean	0.067	0.02	1.41	0.005	0.005	1.0	0.0
Variance	$1\times 10^{-4}$	$2.25\times 10^{-4}$	0.02	$1\times 10^{-4}$	$3.6\times 10^{-5}$	0.01	0.03

**Table 6:** Van Genuchten parameters constraints.

Parameter	$\theta_{r}$	α	*n*	$k_{s}$
Maximum value	0.095	0.075	1.89	0.0737
Minimum value	0.034	0.01	1.23	0.0012

**Table 7:** Validation results. The RMSE values of the corrected sensor readings are reported for the different methods and for varying values of *r* and $\sigma_{obs}$. The best result obtained is highlighted in bold.

*r*	$\sigma_{\mathrm{obs}}$	Corrected RMSE	
		IES	PF
100	0.01	0.0533	0.0309
	0.02	0.0439	0.0340
	0.03	0.0413	**0.0164**
50	0.01	0.0498	0.0398
	0.02	0.0401	0.0438
	0.03	0.0346	0.0372
25	0.01	0.0483	0.0447
	0.02	0.0436	0.0438
	0.03	0.0413	0.0311

**Table 8:** Estimates of the Van Genuchten model parameters, $k_{u}$, and calibration parameters using the IES method with $\sigma_{obs}$ values of 0.01, 0.02 and 0.03 for the application case with field data.

Parameter	$\sigma_{\mathrm{obs}}$	$\theta_{r}$	α	*n*	$k_{s}$	$k_{u}$	*a*	*b*
Initial value		0.067	0.02	1.41	0.005	0.005	1	0
Estimates	0.01	0.0647	0.0012	1.4028	0.0088	0.0053	1.3025	−0.2023
	0.02	0.0678	0.0026	1.4051	0.0095	0.0050	1.1228	−0.1487
	0.03	0.0649	0.0063	1.4060	0.0094	0.0049	1.0311	−0.1236

**Table 9:** Estimates of the Van Genuchten model parameters, $k_{u}$, and calibration parameters using the PF method with $\sigma_{obs}$ values of 0.01, 0.02 and 0.03 for the application case with field data.

Parameter	$\sigma_{\mathrm{obs}}$	$\theta_{r}$	α	*n*	$k_{s}$	$k_{u}$	*a*	*b*
Initial value		0.067	0.02	1.41	0.005	0.005	1	0
Estimates	0.01	0.0741	0.01858	1.4839	0.0020	0.0053	0.5072	0.0623
	0.02	0.0562	0.0192	1.3253	0.0185	0.0052	0.7940	−0.0278
	0.03	0.0672	0.0196	1.4228	0.0040	0.0048	0.9710	−0.1110

**Table 10:** Mean execution time (min) for IES and PF methods across different ensemble sizes, averaged over three observation noise levels.

Method	r	Time (min)
IES	25	22
	50	56
	100	84
PF	25	19
	50	59
	100	104

**Table 11:** Performance of irrigation control under different drift error in sensor readings.

*b*	Running Time (min)	Start-Ups	Water Consumption (L)
−0.12	570	23	4560
0.12	135	5	1080
0.012	255	9	2040
−0.012	270	9	2160
0	255	9	2040

## Data Availability

Data will be made available on request.
